# Anti-parasitic benzoxaboroles are ineffective against *Theileria parva in vitro*

**DOI:** 10.1016/j.ijpddr.2023.10.003

**Published:** 2023-10-10

**Authors:** Pieter C. Steketee, Edith Paxton, Michael P. Barrett, Michael C. Pearce, Timothy K. Connelley, Liam J. Morrison

**Affiliations:** a The Roslin Institute, Royal (Dick) School of Veterinary Studies, University of Edinburgh, Midlothian, EH25 9RG, UK; b Wellcome Centre for Integrative Parasitology, School of Infection and Immunity, College of Medical, Veterinary & Life Sciences, University of Glasgow, Glasgow, G12 8TA, UK; c Global Alliance for Livestock Medicines, Doherty Building, Pentlands Science Park, Edinburgh, EH26 0PZ, UK

## Abstract

East Coast Fever (ECF) is a disease affecting cattle in sub-Saharan Africa, caused by the tick-borne Apicomplexan pathogen *Theileria parva*. The disease is a major problem for cattle farmers in affected regions and there are few methods of control, including a complex infection and treatment vaccine, expensive chemotherapy, and the more widespread tick control through acaricides. New intervention strategies are, therefore, sorely needed. Benzoxaboroles are a versatile class of boron-heterocyclic compounds with demonstrable pharmacological activity against a diverse group of pathogens, including those related to *T. parva*. In this study, the *in vitro* efficacy of three benzoxaboroles against the intracellular schizont stage of *T. parva* was investigated using a flow cytometry approach. Of the benzoxaboroles tested, only one showed any potency, albeit only at high concentrations, even though there is high protein sequence similarity in the CPSF3 protein target compared to other protozoan pathogen species. This finding suggests that benzoxaboroles currently of interest for the treatment of African animal trypanosomiasis, toxoplasmosis, cryptosporidiosis and malaria may not be suitable for the treatment of ECF. We conclude that testing of further benzoxaborole compounds is needed to fully determine whether any lead compounds can be identified to target *T. parva*.

## Graphical abstract

**Figure:**
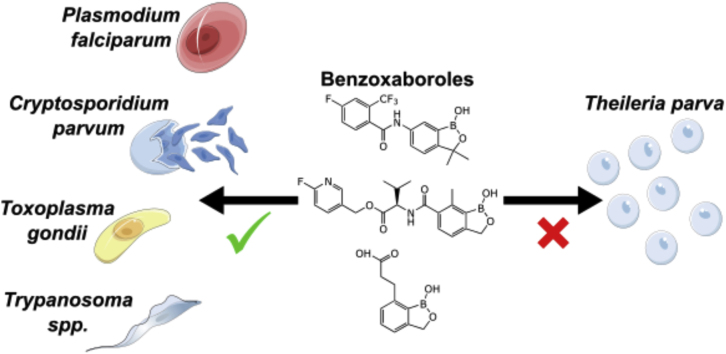

## 1 Introduction

East Coast fever (ECF) is a fatal disease of cattle caused by the Apicomplexan parasite *Theileria parva*, and is prevalent in eastern and southern Africa (Morrison, 2015). The disease is principally transmitted by the brown ear tick *Rhipicephalus appendiculatus* (Bishop et al., 2004). ECF kills approximately 1 million cattle annually, with a further 50 million cattle at risk, resulting in economic losses estimated to be at least $300 million (Nene et al., 2016). An ECF vaccine (ITM) is available but it is expensive and logistically challenging to deploy (Bishop et al., 2020). Therefore, the main method of control for ECF is regular acaricide treatment (vector control) of cattle. Buparvaquone (BPQ), a hydroxynaphthoquinone antitheilerial, is regularly used to treat tropical theileriosis caused by *Theileria annulata* across North Africa and Asia, and has been available for several decades (Kolakowski et al., 2022; McHardy et al., 1985). BPQ can also used as ECF treatment, although it is expensive and not widely used, and needs to be administered early to be efficacious (Muraguri et al., 2006).

The mode of action of BPQ is not fully elucidated and treatment failure due to drug resistance, specifically in *T. annulata,* has been reported (Sharifiyazdi et al., 2012). Analysis of resistant isolates point to mutations in the cytochrome *b* gene, suggesting this may be a site of buparvaquone action (Sharifiyazdi et al., 2012; Hacilarlioglu et al., 2023; Mhadhbi et al., 2015; Sharifiyazdi et al., 2012). Mutations in peptidyl prolyl isomerase PIN1, a secretory factor required to maintain host leukocyte transformation, have also been implicated in BPQ resistance (Marsolier et al., 2015; Salim et al., 2019). A novel, cost-effective chemotherapeutic treatment for ECF with a mode of action distinct from BPQ would be a significant benefit to animal health and food security in affected regions, in particular due to the complexities involved with vaccine use and the potential for BPQ resistance in *T. parva*. Furthermore, the high frequency of acaricide treatment required to control ECF vectors can be toxic to the host and environment, and lead to tick resistance (Githaka et al., 2022). Taken together, these challenges in ECF prevention and management have led to the demand for novel intervention strategies. Indeed, several studies have already utilised open-source compound libraries such as the Medicines for Malaria Venture Pathogen Box with the aim of identifying novel anti-theilerials (Hostettler et al., 2016; Nugraha et al., 2019; Nyagwange et al., 2019; Villares et al., 2022).

Benzoxaboroles are a recently developed class of compounds which exhibit antiviral (Kuhl et al., 2022), antibacterial, antifungal (Rock et al., 2007) and antiparasitic (Giordani et al., 2020; Wall et al., 2018) activities. One of the first benzoxaboroles brought to market, Tavaborole (AN2690), targets the fungal tRNA-leucyl synthetase (Rock et al., 2007). Benzoxaboroles have also been shown to target PDE4 nucleotide phosphodiesterase (Freund et al., 2012) in eukaryotic cells, and β-lactamase (Xia et al., 2011), aminoacyl-tRNA synthetases and carbonic anhydrases (Bonardi et al., 2020) in bacteria. The majority of benzoxaboroles with antiprotozoal potency target the cleavage and polyadenylation specificity factor (CPSF3) (Wall et al., 2018), thus inhibiting mRNA processing (Begolo et al., 2018) and perturbing S-adenosyl-L-methionine metabolism (Steketee et al., 2018). Importantly, these sites of action are unrelated to known buparvaquone targets. Furthermore, benzoxaboroles have shown promising activity against the pathogens that cause Animal African Trypanosomiasis (AAT) (Giordani et al., 2016), a disease that is co-endemic with ECF, and therefore, any compounds that are active both against pathogens that cause ECF and those that cause AAT would be highly desirable.

Recent studies demonstrated that some benzoxaboroles exhibit potency against Apicomplexan pathogens related to *Theileria* spp., including *Plasmodium falciparum* (Sonoiki et al., 2017), *Toxoplasma gondii* (Palencia et al., 2017), *Cryptosporidium parvum* (Lunde et al., 2019) and *Sarcocystis neurona* (Hunt et al., 2021). Importantly, the compounds are able to target these pathogens during intracellular stages of infection. However, the effectiveness of benzoxaboroles against *Theileria* spp. has not been determined *in vitro* or *in vivo*. In this study, we assessed whether three benzoxaborole compounds exhibit *in vitro* potency against *T. parva*. We report very limited potency of any benzoxaborole against *in vitro* cultured intracellular schizont stages of *T. parva*. One of the tested compounds killed parasites at a high micromolar concentration. The other two were ineffective. Analysis of the nucleotide and amino acid sequence of the *T. parva* gene encoding the protozoal CPSF3 target did not highlight any obvious potential resistance determinants. We conclude that further work is required if the benzoxaborole class is to provide novel chemotherapeutics against ECF.

## 2 Materials and methods

### 2.1 Drugs and reagents

Efficacy of three benzoxaboroles, acoziborole, AN11736 and AN3661, was assessed. All compounds were dissolved in DMSO at a concentration of 50 mM except: diminazene aceturate, which was dissolved at a concentration of 200 mM, and AN3661, which was dissolved at a concentration of 10 mM. AN3661 was purchased from GLPBIO (USA). AN11736 was provided by the Global Alliance for Livestock Medicines (GALVmed), and acoziborole was a generous gift from Anacor.

To test drug sensitivity of *T. parva*, a six-point, two-fold dilution series of each compound was used, with initial starting concentrations at 200 nM (lowest concentration: 6.25 nM). In some instances the dosage was increased to 1 μM (AN11736) and 50 μM (acoziborole, AN3661) to test whether increased concentrations reduced *T. parva* viability.

### 2.2 Cell lines

Cell lines infected with *T. parva* (Muguga and Marikebuni strains) were generated by infection of bovine PBMCs as previously described (Goddeeris and Morrison, 1988). The cell lines used in this study, TpMug592 and TpMar, were both infected B cell lines. Cells were maintained in RPMI-1640 supplemented with 10% FCS, 1:100 penicilllin/streptomycin and 2 mM L-glutamine. Cells were incubated at 37 °C and 5% CO_2_. For experiments involving *T. brucei*, the Lister 427 strain was used, and cells were cultured in HMI-11 (Hirumi and Hirumi, 1989) and incubated at 37 °C, 5% CO_2_.

### 2.3 Drug sensitivity assays for *Theileria parva* Muguga and Marikebuni

Drug sensitivity assays for *T. parva* (TpMug592 and TpMar) were carried out using a modified version of a protocol previously developed by Rocchi and colleagues (Rocchi et al., 2008). *T. parva*-infected B-cells were seeded at a density of 1 × 10^5^ cells/mL in 24-well plates and a serial dilution of drug (dissolved in DMSO) was added. Cells were then incubated at 37 °C, 5% CO_2_ for 72 h. Subsequently, cells were harvested and washed in PBS prior to staining with Zombie Violet (Biolegend, USA; 1:1000 dilution in PBS) for 15 min at room temperature (RT), protected from light. Cells were then washed in PBS and fixed for 10 min at RT using 4% paraformaldehyde. Samples were washed in PBS again and subsequently permeabilised (permeabilisation buffer: 5% FBS, 0.02% sodium azide, 0.1% saponin and 20% heat-inactivated goat serum in PBS) for 30 min at RT. After permeabilisation, samples were incubated for 30 min at 4 °C with monoclonal antibody IL-S40.2, which recognises the Polymorphic Immunodominant Molecule (PIM) in *T. parva* (1:4000 dilution of a 2.31 mg/mL stock in permeabilisation buffer) (Rocchi et al., 2008). Cells were washed twice with PBS prior to incubation with a secondary antibody for 30 min at 4 °C (anti-mouse IgG2a AF488, diluted 1:1000 in permeabilisation buffer; Life Technologies). Stained cells were washed twice more in permeabilisation buffer followed by one wash in PBS and a further fixation step with 1% PFA before analysis by flow cytometry.

Sample data were acquired using a BD Fortessa X20 Cell Analyzer (BD Biosciences). A minimum of 10,000 cells were acquired per sample. Data analysis was performed using FlowJo software (BD Life Sciences). Firstly, samples were gated for singlets via forward and side scatter. Live host cells were gated using cell viability staining (Zombie Violet). The percentage of live host cells staining positively for the parasite-specific IL-S40.2 antibody was recorded as an indicator for *T. parva* survival.

### 2.4 Drug sensitivity assays for *Trypanosoma brucei*

To confirm the potency of benzoxaboroles, positive control experiments were carried out using *T. brucei* Lister 427, for which susceptibility to acoziborole and AN11736 is well established (Giordani et al., 2020; Steketee et al., 2018). Standardised alamar blue assays were used to determine EC_50_ of benzoxaboroles, with diminazene aceturate acting as control (Steketee et al., 2018). Briefly, cells at a final density of 2 × 10^4^ cells/mL were incubated in 1:2 serial dilutions (23 × ) of the compounds for 48 h. The starting concentrations were 50 μM (AN3661, acoziborole) or 50 nM (AN11736, diminazene aceturate). Following addition of 20 μL resazurin sodium salt (0.49 mM in 1 × PBS, pH 7.4; Sigma) to each well, plates were incubated for a further 24 h and fluorescence was subsequently measured using a GEN5 Cytation plate reader with the following parameters: *λ*_excitation_ 530 nm and *λ*_emission_ 590 nm.

### 2.5 Analysis of drug sensitivity data

Analyses of drug sensitivity data for both *T. parva* and *T. brucei* were performed using GraphPad Prism (v8.4.0 for Windows, GraphPad Software, CA, USA). Briefly, drug concentrations were transformed to Log_10_ [concentration (M)], and plotted against percentage cells stained positively for IL-S40.2. For experiments involving *T. brucei*, EC_50_ values were calculated using the nonlinear regression and the Sigmoidal dose-response (variable slope) functions in GraphPad Prism.

### 2.6 Sequencing of *T. parva* CPSF3

The mRNA sequence of TpCPSF3 from TpMug592 was sequenced to confirm the isolate used in this study matched the expected sequence of the *T. parva* Muguga reference strain. 1.5 × 10^7^ TpM cells were centrifuged (300×*g*, 5 min) and resuspended in 1 mL TRIzol reagent (Invitrogen). RNA extraction was carried out according to the manufacturer's instructions. Briefly, 200 μL chloroform was added and the sample mixed vigourously. After a 5 min incubation, the sample was centrifuged (12,000×*g*, 15 min, 4 °C) and the aqueous phase was transferred to a fresh 1.5 mL eppendorf tube with 500 μL isopropanol and 1 μL GlycoBlue coprecipitant (Invitrogen). The sample was mixed by vortexing and incubated for 10 min at room temperature prior to a further centrifugation step (12,000×*g*, 15 min, 4 °C). The pellet was washed twice with ice-cold 75% ethanol and dried in a biological safety cabinet. The sample was resuspended in 100 μL nuclease-free water and DNase treated (Turbo DNA-free; Ambion).

Reverse transcription was carried out using the Superscript III kit (Invitrogen), according to the manufacturer's instructions, with a total of 400 ng RNA. The entire TpCPSF3 sequence (2127 bp) was amplified (forward primer: 5′-ATGGATGATAGAGTTAGAAT; reverse primer: 5′- TTATATTAAAATATGATTGGACGT) using the Q5 Hot-Start High-Fidelity 2 × master mix (New England Biolabs) according to the manufacturer's instructions (4 μL cDNA in a 50 μL reaction), with the following PCR cycling parameters: 98 °C, 30 s; 35 cycles of 98 °C, 10 s, 55 °C, 30 s, 72 °C, 1 min 54 s; and a final 10 min extension at 72 °C. The PCR product was run on a 1% agarose gel and the band corresponding to the CPSF3 gene was excised and purified using a Gel Extraction kit (QIAgen).

The TpCPSF3 gene was sequenced (DNA Sequencing and Services, University of Dundee) using the aforementioned primers as well as the following primers: fwd1: GGAGCTGGTTGTGAAGTTGG, fwd2: TGCGTAATGGGAAGTGTCTT, fwd3: ACCATTGGATCACTCTCATCAGA, rev1: ACTCGTCACTAACTTGATCACC, rev2: AGTTCATCAGCCAGAGTTCCT, rev3: TGTCATTAACGGTAATCTCCTGA. Sequencing data was analysed using CLC Main Workbench (v22.0.2, QIAgen).

### 2.7 Protein alignments

For protein alignments, the CPSF3 protein sequence from *T*. *parva* (Gene ID: TpMuguga_03g00560), *Theileria annulata* (strain Ankara, gene ID: TA05230), *Babesia bigemina* (strain BOND, gene ID: BBBOND_0405580), *P*. *falciparum* (strain 3D7, gene ID: PF3D7_1438500), *T*. *gondii* (strain ME49, gene ID: TGME49_285200), *C. parvum* (strain Iowa II, gene ID: cdg_460), *T*. *brucei brucei* (strain Lister 427, gene ID: Tb927.4.1340), *Trypanosoma congolense* (strain IL3000, gene ID: TcIL3000_4_840) and *Homo sapiens* (gene ID: ENSG00000119203) were obtained from the VEuPathDB Bioinformatics Resource Centre (release v60.0) and associated portals TriTrypDB, PlasmdoDB, ToxoDB, CryptoDB, PiroplasmaDB and HostDB (Amos et al., 2022).

Domain searches were performed using the SMART database (Letunic et al., 2021). Alignments were generated and sequence similarities were calculated using CLC Main Workbench v22.0.2. Figures were generated using a combination of CLC Main Workbench, Inkscape and Microsoft Powerpoint.

To confirm whether or not cysteine peptidases required for activation of the AN11736 pro-drug were encoded by *T. parva*, the relevant cysteine peptidase gene and protein sequences were obtained from the *T. brucei* Lister 427 genome (gene IDs: Tb927.10.1030, Tb927.10.1040 and Tb927.10.1050) and searched for in the *T. parva* Muguga genome by BLAST-P in PiroplasmaDB.

## 3 Results and discussion

### 3.1 Analysis of *T. parva* sensitivity to benzoxaborles

**Fig. 1:**
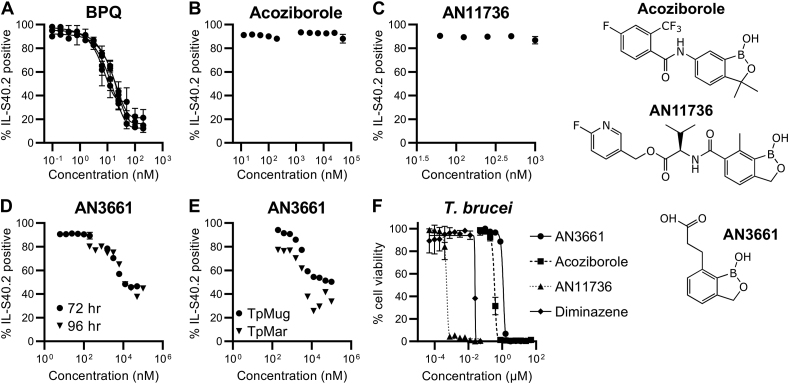
**Drug sensitivity assays of benzoxaboroles in *T. parva*-infected B-cells.** A flow-cytometry-based protocol was developed to test drug efficacy against the intracellular schizont stage of *T. parva in vitro*, using Buparvaquone (A) as a control. Compounds were added in a serial dilution, and *T. parva* viability was interpreted as a function of percentage (%) IL-S40.2 positive staining. (B–E) The potency of three benzoxaboroles were subsequently tested, highlighting a lack of *T. parva* killing when treated with Acoziborole and AN11736. (D) When used at higher concentrations (>1 μM) AN3661 led to a reduced percentage of IL-s40.2-positive cells. Extending the experiment by 24 h (96 h total) did not lead to further reductions in percentage IL-s40.2 positive cells. (E) High doses of AN3661 were subsequently tested on a different strain of *T. parva* (TpMar) to confirm the impact of this benzoxaborole was not strain-specific. Benzoxaborole activity was confirmed for each compound using an *in vitro* model of *T. brucei* (F). Structures for the relevant benzoxaboroles are shown to the right of the panels. Abbreviations: BPQ: buparvaquone.

**Fig. S1:**
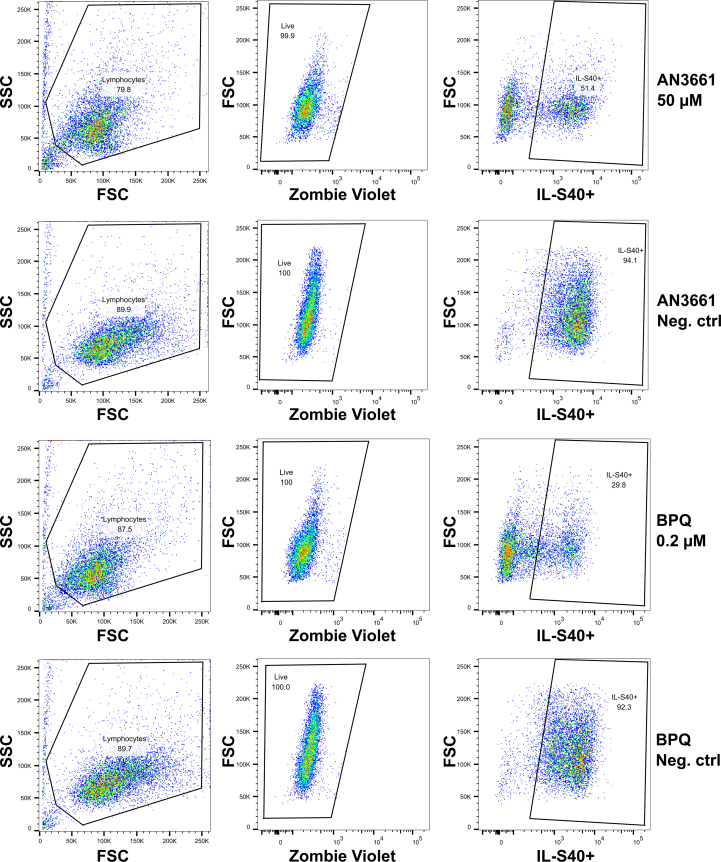
**flow cytometry analysis of *T. parva* drug sensitivity.** Example Flow charts showing the gating strategies used for *T. parva* drug sensitivity assays. Singlet cells were selected via forward and side scatter. Live host cells were then gated using a live/dead stain (Zombie Violet). Finally, host cells containing live *T. parva* schizonts were gated using the IL-S40+ stain, specific to the *T. parva* PIM protein. Plots are shown for experiments involving both AN3661 and BPQ alongside negative controls as indicated.

To determine whether benzoxaboroles exhibit potency against *T. parva*, we modified a previously described drug sensitivity assay using a flow cytometry-based approach (Rocchi et al., 2008). To test this approach, we carried out drug sensitivity assays using the commercially available antitheilerial buparvaquone (Fig. 1A & Fig. S1) and *T. parva* cell line TpMug592 (see Methods). Using this assay we observed clear killing of *T. parva*-infected cells by buparvaquone, and obtained an EC_50_ of 12.7 ± 2.4 nM. This value is higher than previously reported values of 2 nM (Hostettler et al., 2016) and 4.2 nM (Nyagwange et al., 2019), but lower than another reported value of 153 nM (Barman et al., 2021). This variation is very likely due to differences in the experimental procedure, and in particular the use of *T. annulata* instead of *T. parva* in the latter study.

We next carried out drug sensitivity assays using the optimised assay for three selected benzoxaboroles: AN11736, previously shown to have good activity against African trypanosomes; AN5568 (acoziborole), currently in clinical trials to treat Human African Trypanosomiasis; and AN3661, a potent anti-Apicomplexan with validated efficacy against *Plasmodium falciparum*, *Toxoplasma gondii* and *Cryptosporidium* spp. At starting concentrations of 200 nM we detected no change in percentage of cells with viable *T. parva* schizonts for AN5568 (acoziborole; Fig. 1B) or AN11736 (Fig. 1C). Increasing the concentration of acoziborole to 50 μM had no effect on *T. parva* staining (Fig. 1B).

One compound, AN3661, demonstrated some effect on *T. parva* infected cells when used at a micromolar dose, but also displayed an unusual staining pattern in which ∼40% of cells remained positive for IL-S40.2 irrespective of drug dosage when used above 12.5 μM (Fig. 1D). Extending the incubation time to 96 h prior to analysis did not increase the proportion of cells killed (Fig. 1D). To confirm this finding, further analysis was undertaken with high doses (>1 μM) of AN3661 using a different strain of *T. parva* – Marikebuni (TpMar). AN3661-treated TpMar exhibited a similar reduction in IL-s40 positive cells after a 72 h incubation when compared to TpMug592, indicating that the effect was not strain-specific (Fig. 1E).

Importantly, *T. b. brucei* exhibited sensitivity to all benzoxaboroles (Fig. 1F), with EC_50_s of 1.07 μM, 336.70 nM, 0.49 nM and 24.36 nM recorded for AN3661, acoziborole, AN11736 and diminazene, respectively. Values recorded for acoziborole, AN11736 and diminazene were consistent with previously reported values (Giordani et al., 2020; Steketee et al., 2018, 2021).

### 3.2 *In silico* analysis of benzoxaborole protein targets

**Fig. 2:**
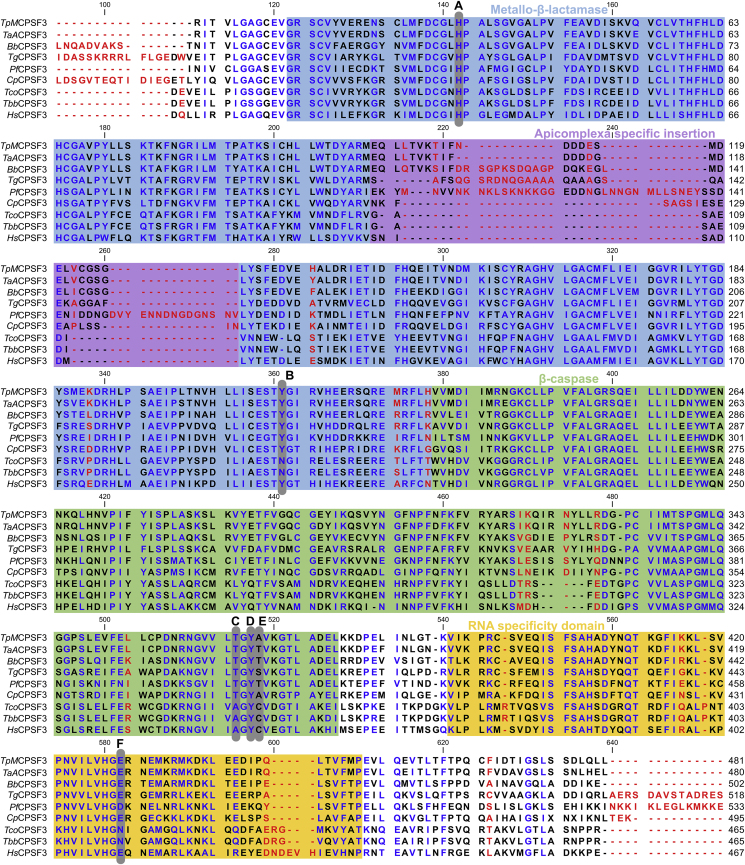
**Multiple sequence alignment of pathogen CPSF3 proteins.** Partial sequences (lacking the CPSF3 C-terminus) are shown and were acquired from pathogen-specific databases (Methods), with colouring based on previously published data (Bellini et al., 2020). Conservation is indicated by letter colouring (red, 0–33%; black, 33–66%; blue, 66–100%) and conserved CPSF3 domain architecture is indicated by background colours as follows: blue, metallo-β-lactamase; purple, apicomplex specific domain; green, β-caspase; yellow, RNA specificity domain. Known amino acid changes that result in benzoxaborole resistance are shown by grey bars: A, H^36^Y (Sonoiki et al., 2017); N^232^H (Wall et al., 2018) and Y^328^H (Palencia et al., 2017); C, T^406^I; D, Y^408^S; E, T^409^A; F, D^470^N (Sonoiki et al., 2017); F, E^545^K (Palencia et al., 2017). (For interpretation of the references to colour in this figure legend, the reader is referred to the Web version of this article.)

Given the observed lack of activity of all three benzoxaboroles against *T. parva*, we elected to focus on the CPSF3 protein target of this class of compounds. Firstly, the CPSF3 gene in the TpMug592 line was sequenced from reverse-transcribed mRNA, in order to confirm that the expressed CPSF3 sequence matched that of the *T. parva* Muguga reference genome (Fig. S2). Once confirmed, CPSF3 protein sequences in all relevant protozoan species were analysed (60% sequence containing the major functional domains shown in Fig. 2). Overall, there were high levels of sequence conservation in CPSF3 domains across all species (Fig. 2), including 92.9% sequence identity between *T. parva* and *T. annulata*, although this conservation was reduced when taking into account the 3’ end of the CPSF3 sequence.

We next assessed whether amino acid differences at sites implicated in benzoxaborole binding may underlie the low observed potency of the benzoxaboroles in *T. parva*, by analysing resistance loci previously described in *P. falciparum*, *T. gondii*, *T. brucei* and *T. congolense*. However, in all but one case (*Pf*CPSF3-T^409^A; equivalent to A^373^ in *T. parva*; Fig. 2, E), all amino acids at these sites were the same as wild-type, i.e. drug sensitive, in other organisms tested. Whilst the A^373^ site could potentially explain reduced sensitivity to AN3661 in *T. parva*, this site is also mutated to alanine in *C. parvum*, which exhibits a sub-micromolar EC_50_ to AN3661 (Swale et al., 2019). It is therefore unlikely that this mutation alone could explain the lack of benzoxaborole activity in *T. parva*.

## 4 Discussion

East Coast fever remains a significant hindrance to sustainable cattle farming in Africa. New chemotherapeutics are sorely needed to target *Theileria parva*, the Apicomplexan parasite that causes ECF (Nyagwange et al., 2019; Villares et al., 2022). Benzoxaboroles are a relatively new class of boronic acid-containing compounds that exhibit high potency against a number of pathogens, in particular important protozoan parasites of humans and animals (Giordani et al., 2016, 2020), including Apicomplexan parasites related to *T. parva*. Several lead benzoxaborole candidates target the pathogen CPSF3 protein, inhibiting mRNA maturation (Begolo et al., 2018; Bellini et al., 2020; Palencia et al., 2017; Wall et al., 2018). However, it is currently unknown whether any benzoxaboroles are efficacious against *T. parva* and the basis of a viable ECF treatment.

In this study, we show that only one of the three benzoxaboroles tested exhibited any potency against intracellular *T. parva* schizonts*,* and its efficacy was marginal – only showing activity at micromolar concentrations. The lack of activity observed for all three benzoxaboroles is surprising, not least in the case of AN3661 – a candidate shown to target the intracellular stages of related Apicomplexan parasites *Plasmodium* spp. and *Toxoplasma gondii* (Palencia et al., 2017; Sonoiki et al., 2017). The lethal dosage for this compound is in the nanomolar range for *P. falciparum* (EC_50_: 32 nM; Sonoiki et al., 2017) and *C. parvum* (EC_50_: 80 nM; Swale et al., 2019), with a higher lethal dose (EC_50:_ 0.9 μM; Palencia et al., 2017) reported for *T. gondii*. Even at the latter concentration, *T. parva* viability is equal to untreated controls, with a reduction in viability only observed at AN3661 concentrations >6 μM. Alignment of CPSF3 protein sequences, in addition to sequencing the TpMug592 CPSF3 gene to confirm sequence identity, did not uncover obvious explanations for this reduction in benzoxaborole activity in *T. parva*. Alternative approaches such as *in silico* drug-protein docking studies could yield further insights into the structure-activity relationship of this class of compounds and the CPSF3 target in *Theileria*.

There are several potential underlying reasons for the observed lack of benzoxaborole activity against *T. parva,* even at higher compound doses. Firstly, the parasite line used resides within B cells *in vitro*. In contrast, *P. falciparum* was maintained in red blood cells (Sonoiki et al., 2017), *T. gondii* was maintained in HFF cells (Palencia et al., 2017), and *C. parvum* was maintained in HCT-8 cells (Swale et al., 2019) in the respective benzoxaborole studies on these parasites. To our knowledge, uptake, metabolism and efflux of benzoxaboroles in lymphocytes has not been studied, although this class of compounds exhibit no genetic toxicity in human peripheral blood lymphocytes (Ciaravino et al., 2013). It is therefore unknown whether this host cell type contains features that may influence benzoxaborole activity and further work, including mass spectrometry analysis, would aid in confirming benzoxaborole uptake both into host cells and schizonts. Similarly, pertinent biological knowledge of *T. parva* is lacking. For example, it is not known whether there are aspects of parasite biology, specifically in the schizont stage, that may impact upon drug uptake, metabolism and kinetics. *T. parva* schizonts are typically multinucleated, in some cases with 30–40 nuclei per schizont (Nene et al., 2016; Shaw, 2003). Given CPSF3 localises to the nucleus, it is possible that only schizonts with low numbers of nuclei are affected by AN3661 treatment, leading to the observed persistence of infected host cells. Furthermore, it is unknown whether the protein target, CPSF3, is essential for *T. parva* schizonts, and this will continue to be difficult to discern in the absence of tools to genetically modify the parasite.

AN11736 exhibits high potency against African trypanosome species. However, this compound did not kill *T. parva*. AN11736 is a pro-drug that requires serine peptidase (CBP)-cleavage to become active (Giordani et al., 2020; Padilla et al., 2022). We were unable to identify a CBP orthologue in the available genome of *T. parva*, nor were we able to identify any gene containing the relevant predicted domain (Pfam: PF00450; InterPro: IPR001563), which may explain why this compound had no effect on *T. parva*. Furthermore, potential host cell-mediated cleavage of this benzoxaborole could also preclude entry into the parasite. We cannot rule out that the tested benzoxaboroles may kill *T. parva* at micromolar concentrations, but they are known to be active against trypanosomes at sub-nanomolar concentrations. Thus, our results suggest that benzoxaborole compounds used at doses effective against AAT are unlikely to be useful for the treatment and control of ECF. Even if effective against *T. parva* at micro- or millimolar concentrations, the amount necessary for treatment of cattle may be prohibitively expensive.

Further work is required to determine whether any benzoxaboroles can effectively target *T. parva,* as well as the closely related *T. annulata*. Given the sequence homology of CPSF3 between *T. parva* and *T. annulata*, we predict that the benzoxaboroles tested in this study would similarly have no effect on the latter. Other benzoxaboroles such as AN2690 and AN2728 are known to target leucyl-tRNA synthetase and phosphodiesterase 4, respectively, in other organisms (Freund et al., 2012; Rock et al., 2007), but whether orthologues of these proteins could be targeted in *T. parva* is currently unknown. Furthermore, drug sensitivity was assayed only in schizonts in this study, and other life stages (e.g. the extracellular sporozoites) should also be analysed. Finally, it is not known whether benzoxaborole efficacy could be affected by host cell type. Such information will be crucial to determine whether this class of compounds may yield sorely needed chemotherapeutics to treat ECF.

## Conflicts of interest

Pieter Steketee, Edith Paxton, Michael Barrett, Timothy Connelley and Liam Morrison: Declarations of interest: none.

Michael Pearce is an employee of the Global Alliance for Livestock Veterinary Medicines (GALVmed), who provided access to one of the test compounds, AN11736.

## Appendix A Supplementary data

Fig. S2 **Sequence chromatograms of TpMug925 CPSF3 cDNA.** The CPSF3 gene was amplified from TpMug592 cDNA and Sanger sequenced using eight primers (indicated by green arrows above the reference sequence). Sanger sequences were aligned using CLC Main workbench to confirm that it matched the annotated CPSF3 sequence in the *T. parva* Muguga reference genome. Fig. S2
