# Supplementary figures and images for: Anti-parasitic benzoxaboroles are ineffective against Theileria parva in vitro
Source: Int J Parasitol Drugs Drug Resist. 2023 Oct 10;23:71–7. doi: 10.1016/j.ijpddr.2023.10.003 (PMC10623109; doi:10.1016/j.ijpddr.2023.10.003)

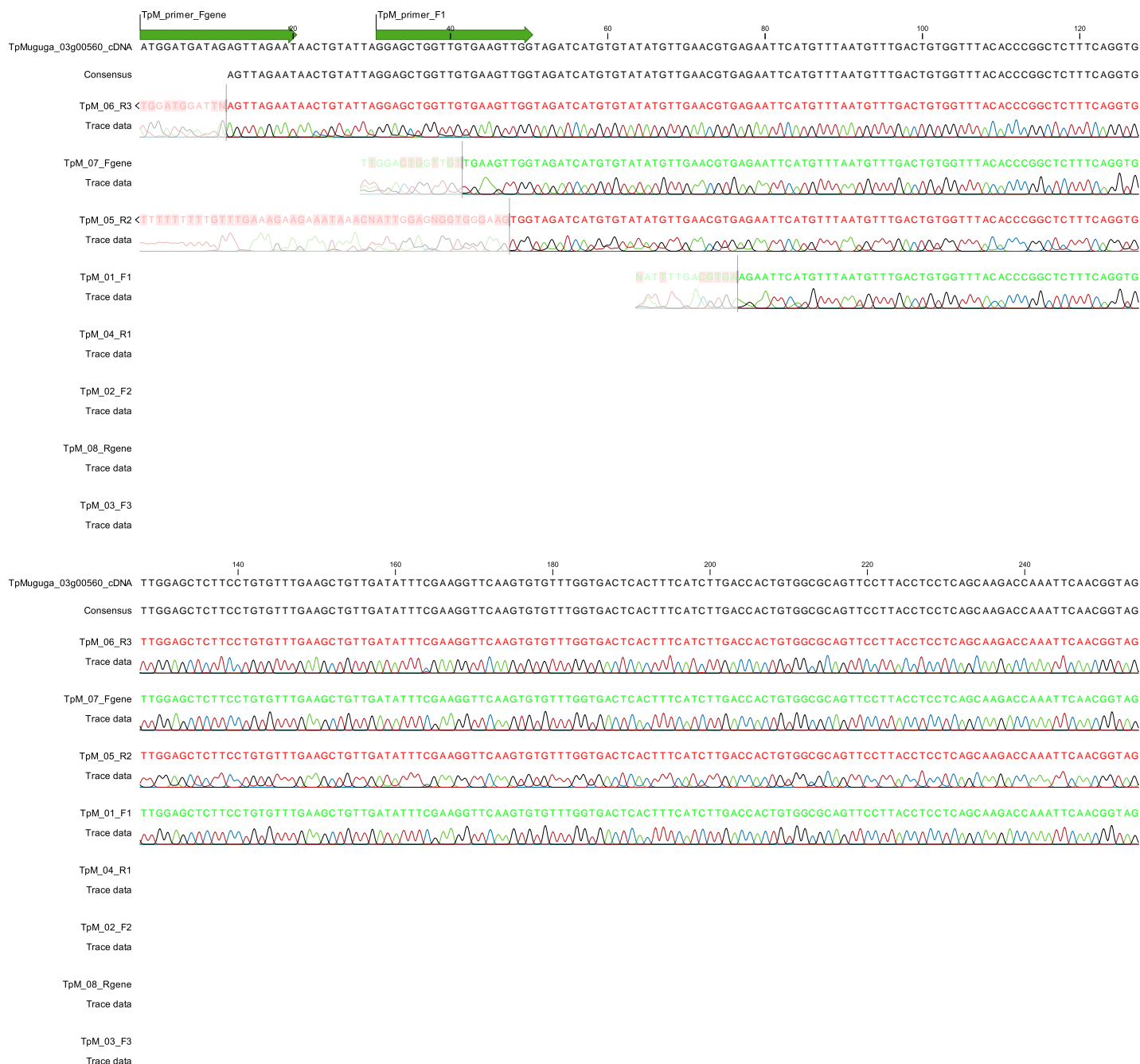

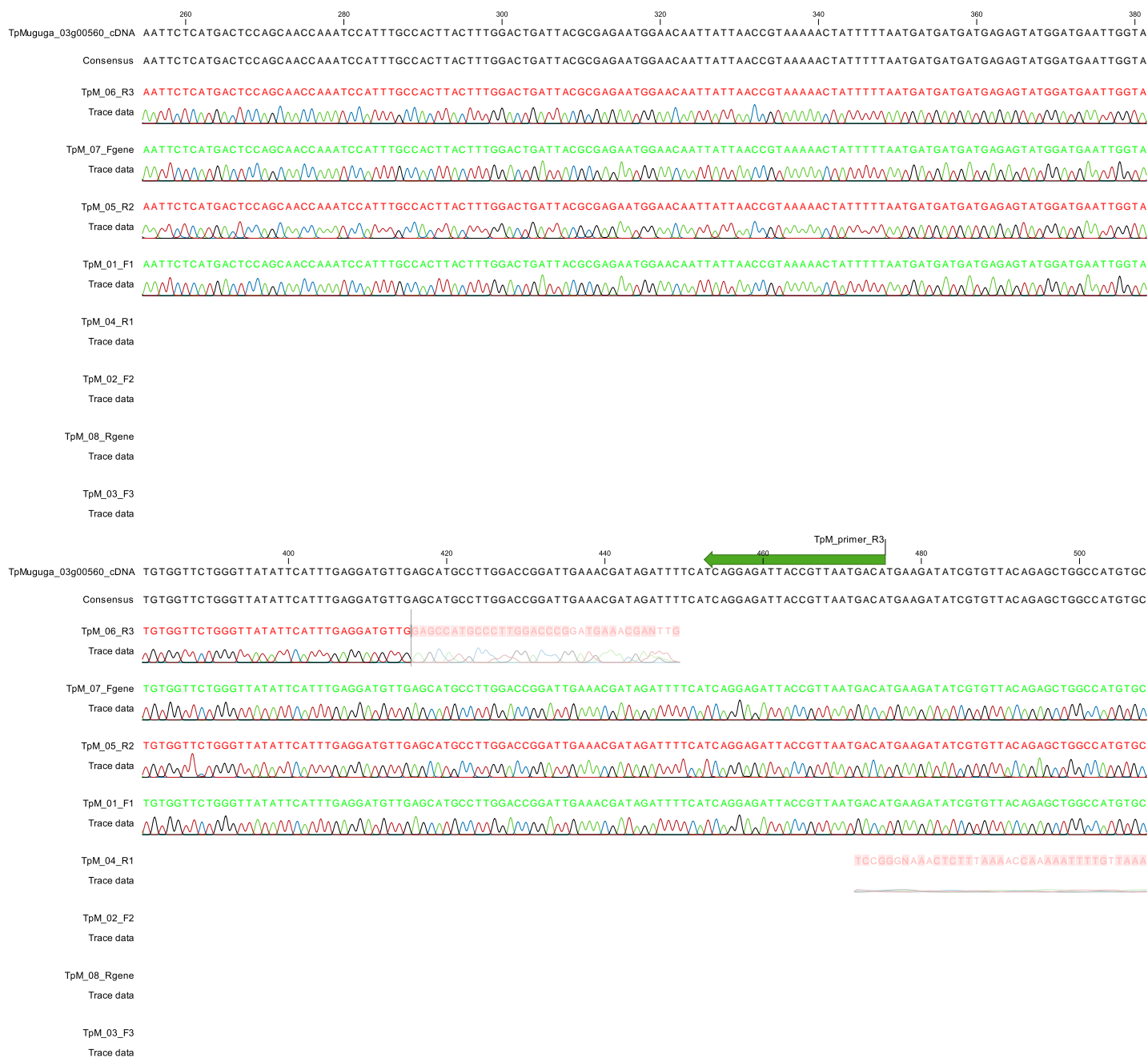

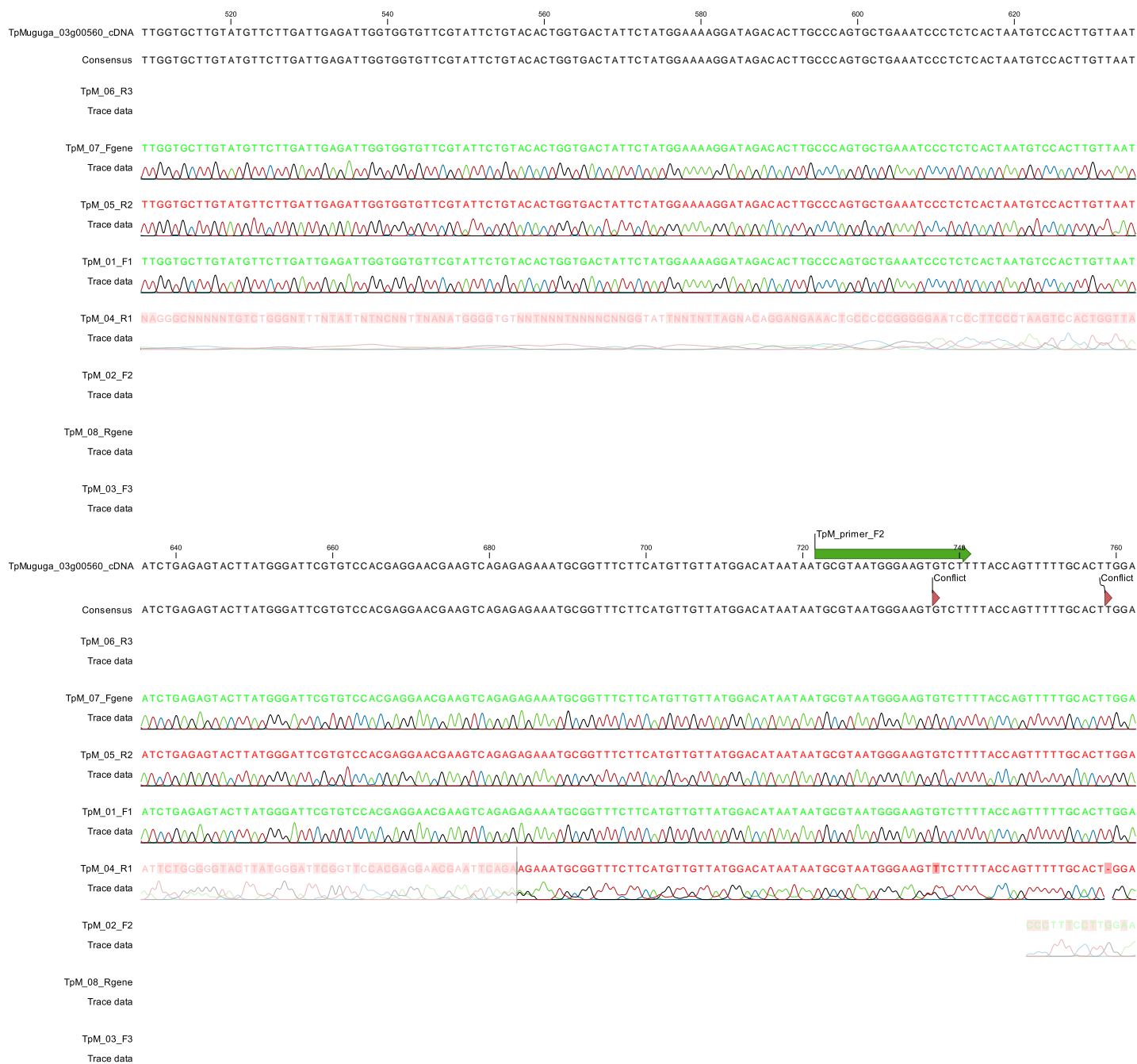

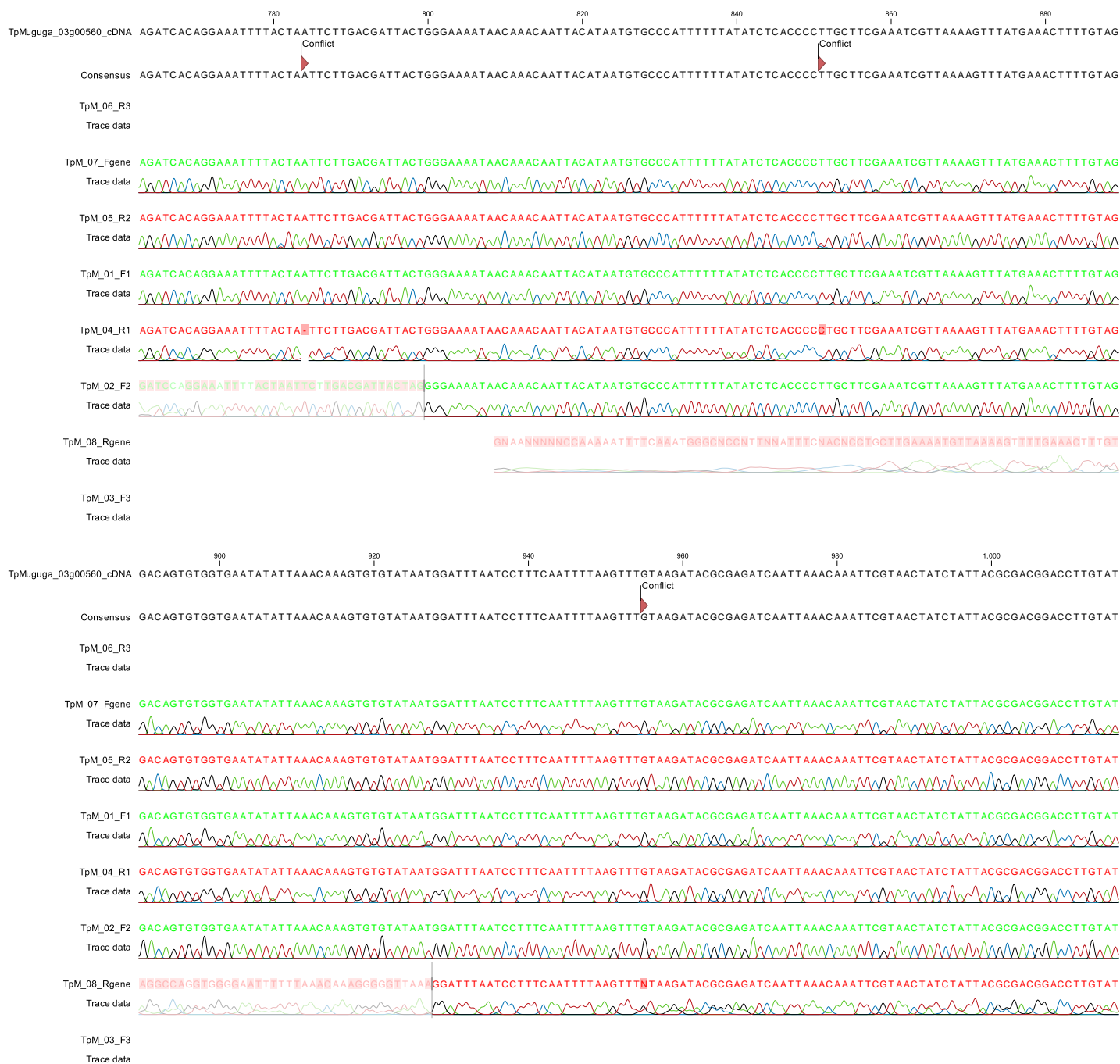

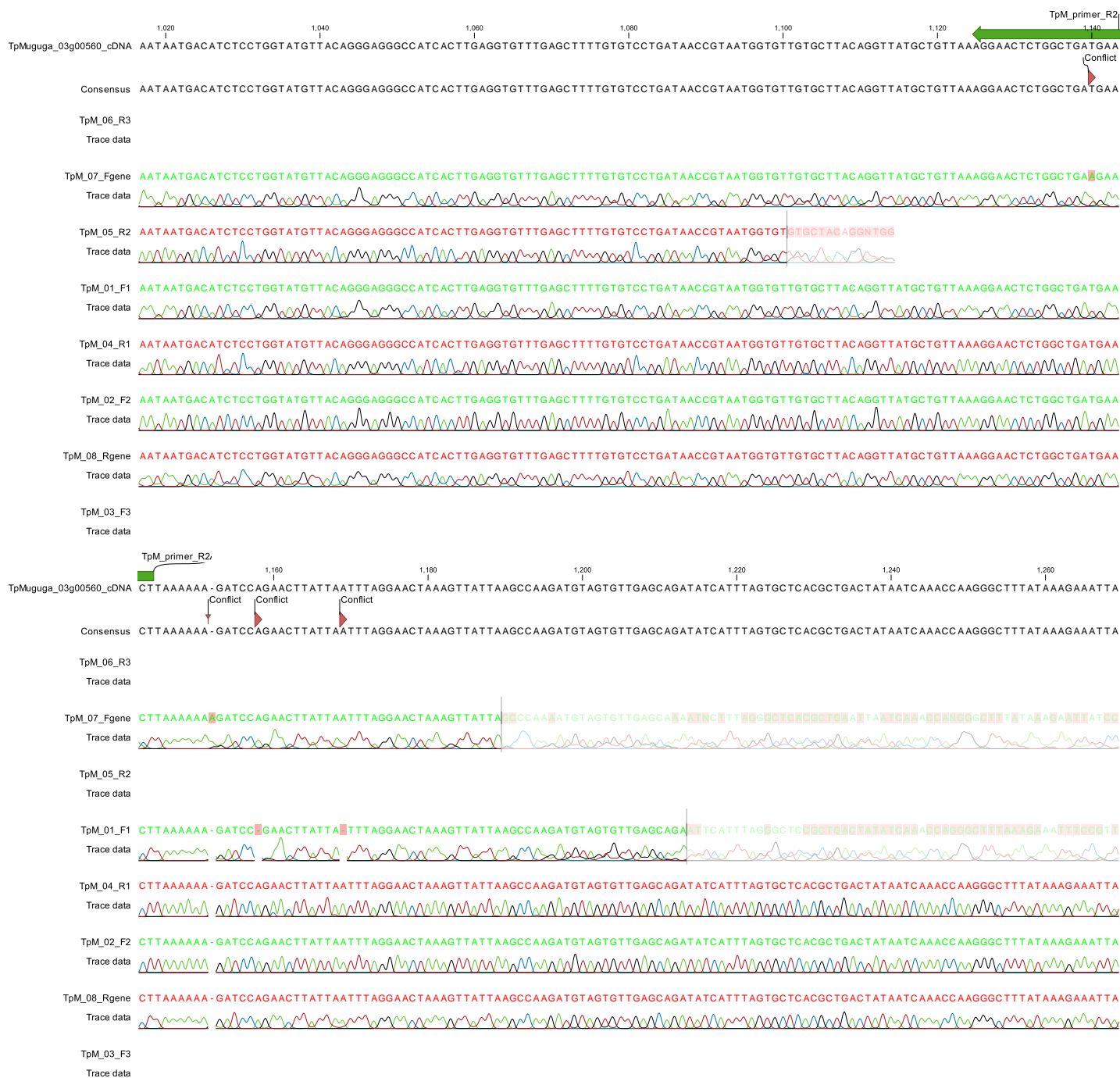

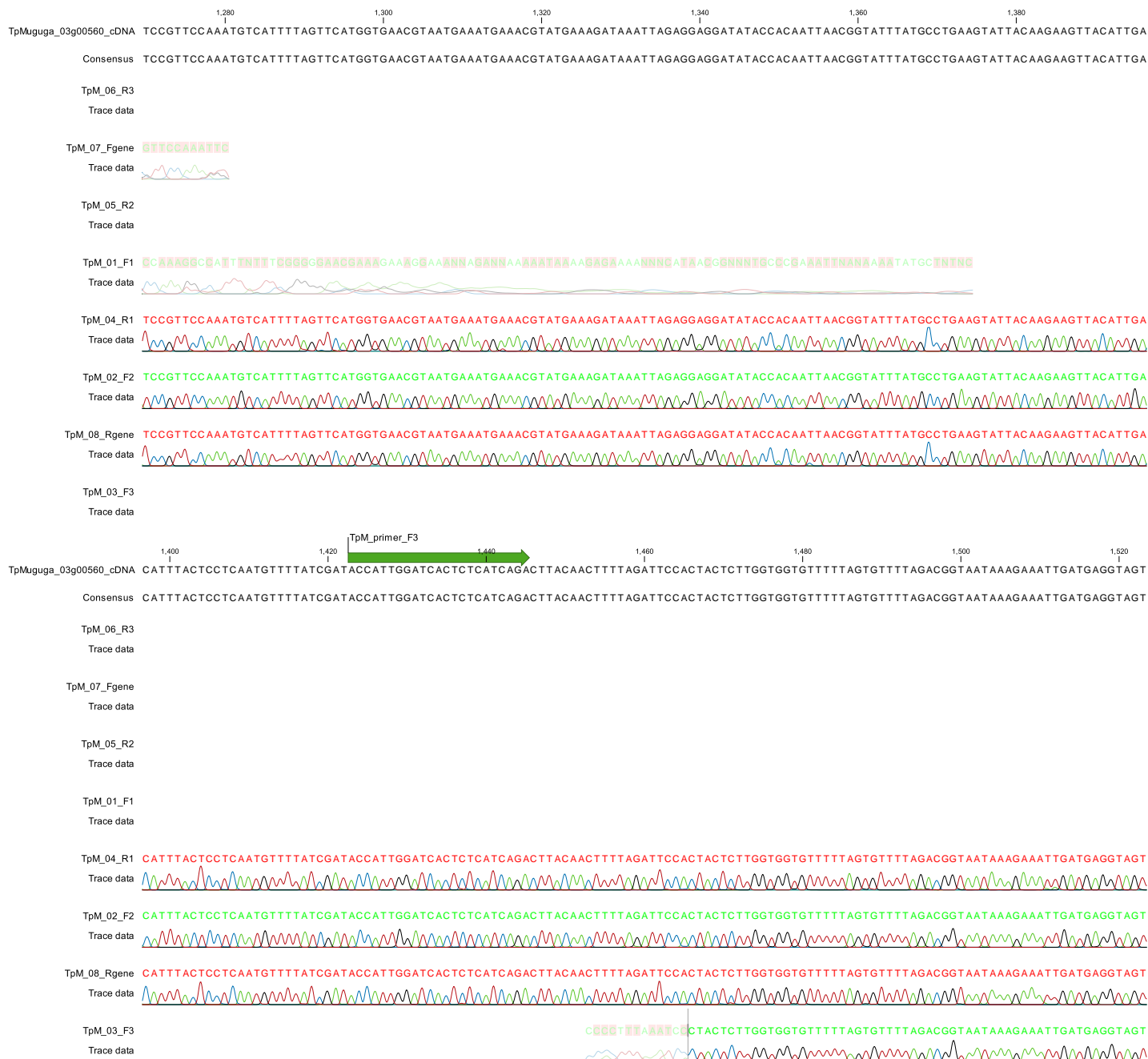

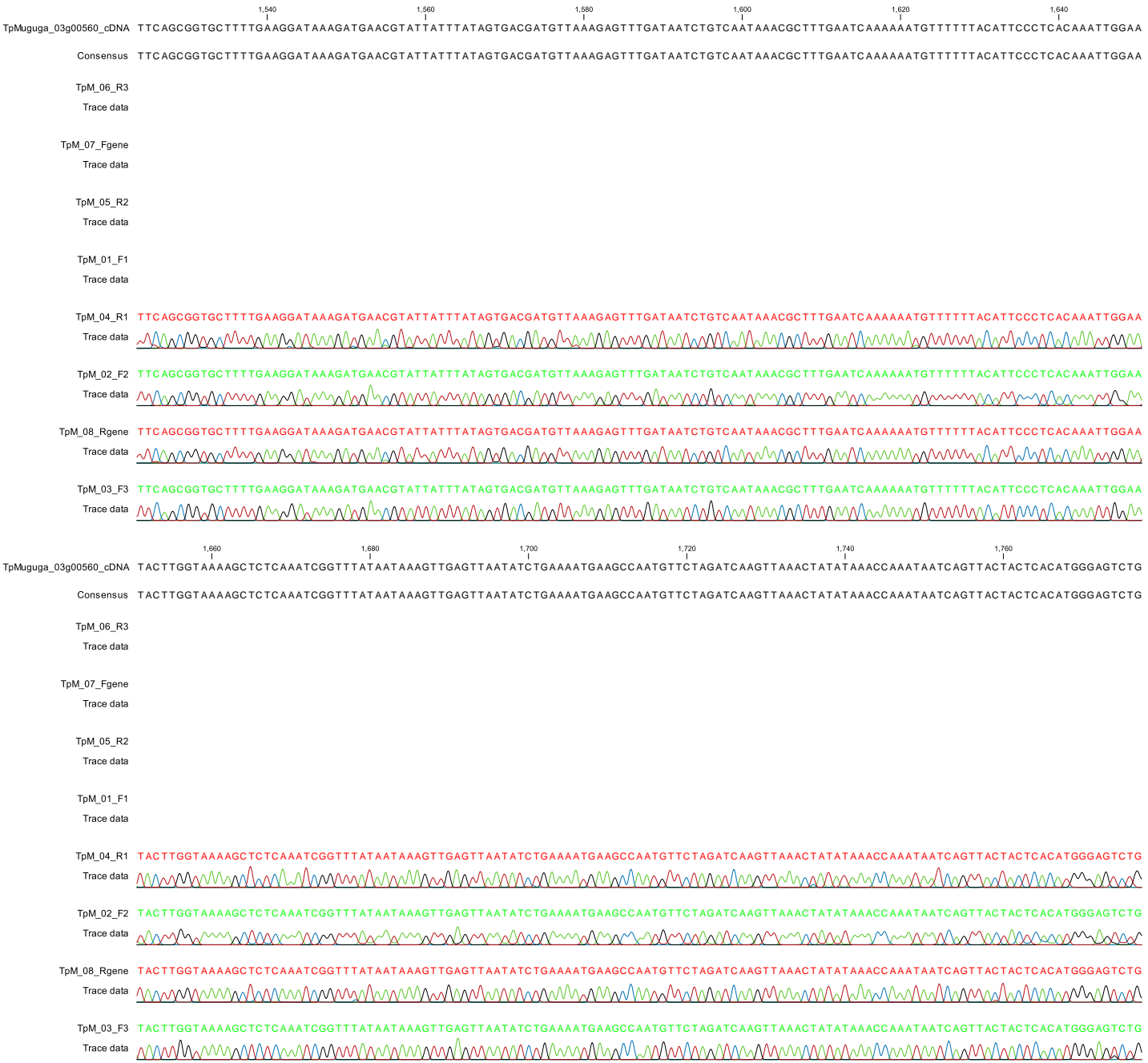

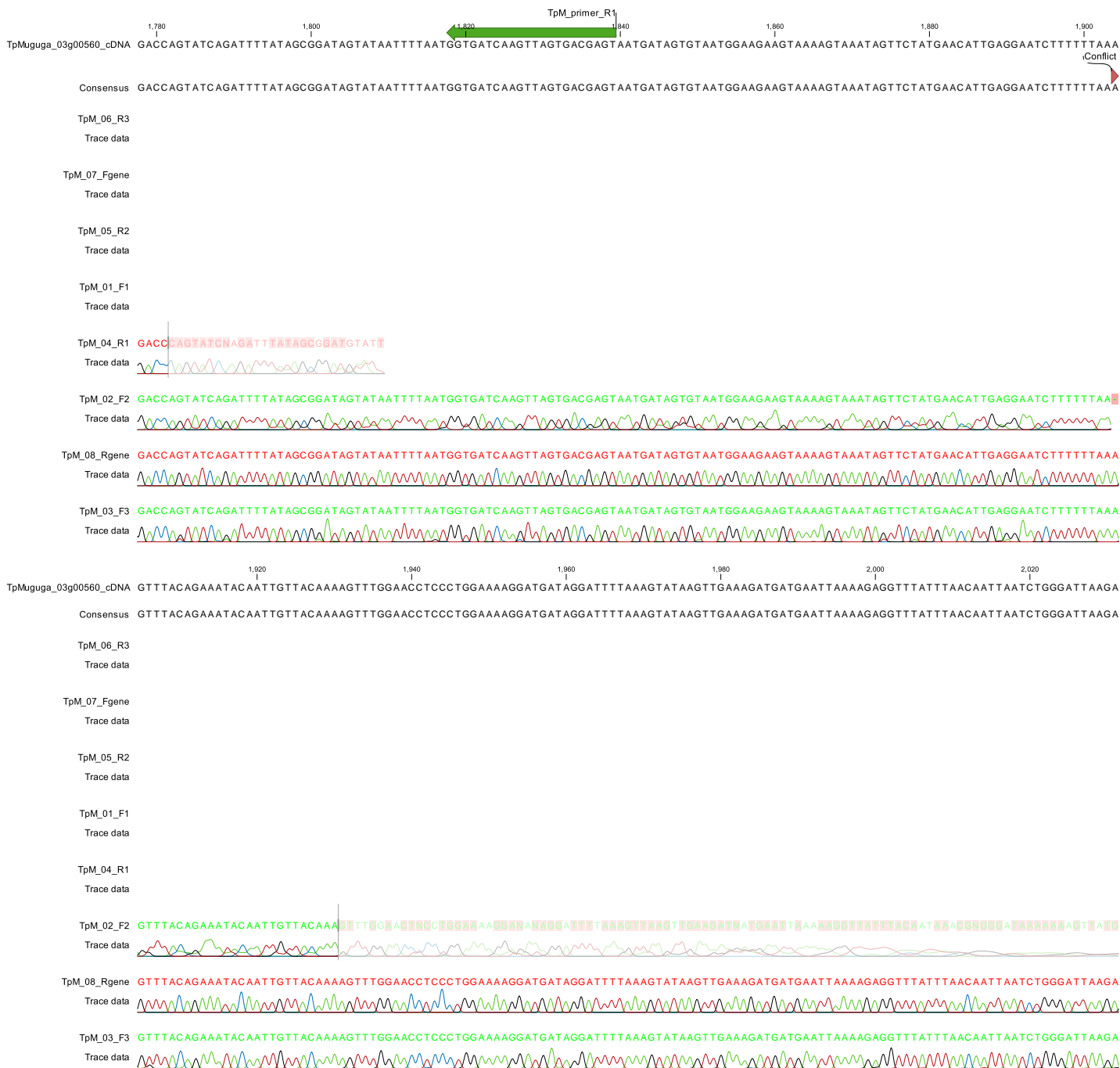

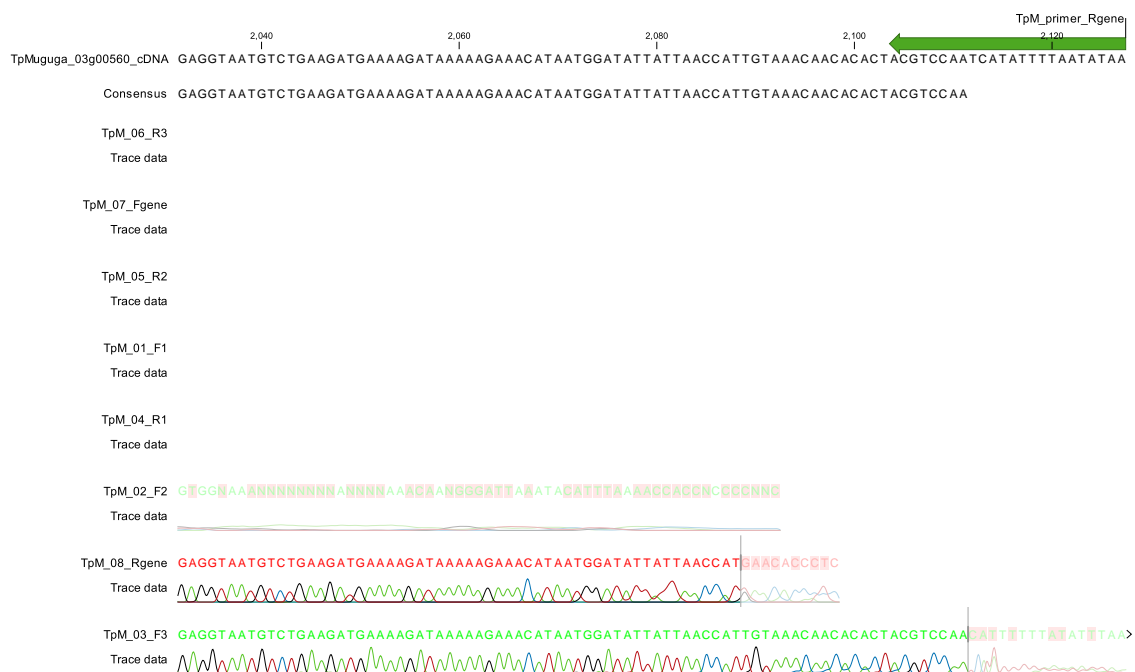

Supplement: Fig. S2 — Sequence chromatograms of TpMug925 CPSF3 cDNA. The CPSF3 gene was amplified from TpMug592 cDNA and Sanger sequenced using eight primers (indicated by green arrows above the reference sequence). Sanger sequences were aligned using CLC Main workbench to confirm that it matched the annotated CPSF3 sequence in the T. parva Muguga reference genome. [file mmc1.pdf]
